# Standardization of the NEO-PI-3 in the Greek general population

**DOI:** 10.1186/s12991-014-0036-9

**Published:** 2014-12-05

**Authors:** Konstantinos N Fountoulakis, Melina Siamouli, Stefania Moysidou, Eleonora Pantoula, Katerina Moutou, Panagiotis Panagiotidis, Marina Kemeridou, Eirini Mavridou, Efimia Loli, Elena Batsiari, Antonio Preti, Leonardo Tondo, Xenia Gonda, Nisreen Mobayed, Kareen Akiskal, Hagop Akiskal, Paul Costa, Robert McCrae

**Affiliations:** Third Department of Psychiatry, School of Medicine, Aristotle University of Thessaloniki, Thessaloniki, Greece; Research associate Aristotle University of Thessaloniki, Thessaloniki, Greece; Department of Psychiatry, 424 Military Hospital, Thessaloniki, Greece; Mental Health Hospital of Thessaloniki, Thessaloniki, Greece; Psychologist in private practice, Athens, Greece; Center of Liaison Psychiatry and Psychosomatics, University Hospital, University of Cagliari, Italy, and Centro Medico Genneruxi, Cagliari, Italy; Mood Center LucioBini, Cagliari and Rome, Italy; McLean Hospital, Harvard Medical School, Boston, USA; Department of Clinical and Theoretical Mental Health, Faculty of Medicine, Semmelweis University, Budapest, Hungary; Department of Psychiatry, University of California, San Diego, California USA; Studies of Temperament and Creativity, Paris, France; International Mood Center, University of California, San Diego, CA USA; Laboratory of Behavioral Neuroscience Biomedical Research Center, Baltimore, MD USA; ᅟ, Baltimore, MD USA

**Keywords:** Five-factor personality inventory, NEO-PI-3, Standardization, Psychometrics

## Abstract

**Background:**

The revised NEO Personality Inventory (NEO-PI-3) includes 240 items corresponding to the Big Five personality traits (Extraversion, Agreeableness, Conscientiousness, Neuroticism, and Openness to Experience) and subordinate dimensions (facets). It is suitable for use with adolescents and adults (12 years or older). The aim of the current study was to validate the Greek translation of the NEO-PI-3 in the general Greek population.

**Material and methods:**

The study sample included 734 subjects from the general Greek population of whom 59.4% were females and 40.6% males aged 40.80 ± 11.48. The NEO-PI-3 was translated into Greek and back-translated into English, and the accuracy of the translation was confirmed and established. The statistical analysis included descriptive statistics, confirmatory factorial analysis (CFA), the calculation of Cronbach’s alpha, and the calculation of Pearson product–moment correlations. Sociodemographics groups were compared by ANOVA.

**Results:**

Most facets had Cronbach’s alpha above 0.60. Confirmatory factor analysis showed acceptable loading of the facets on their own hypothesized factors and very good estimations of Cronbach’s alphas for the hypothesized factors, so it was partially supportive of the five-factor structure of the NEO-PI-3.The factors extracted with Procrustes rotation analysis can be considered reasonably homologous to the factors of the American normative sample. Correlations between dimensions were as expected and similar to those reported in the literature.

**Discussion:**

The literature suggests that overall, the psychometric properties of NEO-PI-3 scales have been found to generalize across ages, cultures, and methods of measurement. In accord with this, the results of the current study confirm the reliability of the Greek translation and adaptation of the NEO-PI-3. The inventory has comparable psychometric properties in its Greek version in comparison to the original and other national translations, and it is suitable for clinical as well as research use.

## Introduction

The NEO Inventories were developed by Paul T. Costa, Jr. and Robert R. McCrae. Because it assessed Neuroticism, Extraversion, and Openness to experience, its original version, developed in 1978, is known as the NEO inventory (NEO-I). The NEO-I measured only three of the Big Five personality traits [1] and was subsequently revised in 1985 to include all five traits under the new title ‘NEO Personality Inventory (NEO-PI).’ It was further refined as the NEO-PI-R [2]. Its latest version is the NEO-PI-3 [3].

The NEO-PI-3 includes 240 items corresponding to the Big Five personality traits (Extraversion, Agreeableness, Conscientiousness, Neuroticism, and Openness to xperience) and subordinate dimensions (facets). It is suitable for use with adolescents and adults (12 years or older). Item responses are made on a five-point scale, ranging from ‘strongly disagree’ to ‘strongly agree’. Electronic and print forms of the inventories are available. Administration of the full version of the NEO-PI-3 takes between 30 and 40 min. Assessment should not be evaluated if there are more than 40 items missing.

The aim of the current study is to validate the Greek translation of the NEO-PI-3 in the general Greek population.

## Material and methods

The study sample included 734 subjects from the general Greek population (436 females, 59.4%; 298 males, 40.6%). Their mean age was 40.80 ± 11.48 years (range 25–67 years): 39.43 ± 10.87 years (range 25–65 years) for females and 42.82 ± 12.06 years (range 25–67 years) for males.

The NEO-PI-3 was translated into Greek by KNF and back-translated into English by two other authors (MS and KM). The originators of the instrument and KNF verified the accuracy of the translation and its conformity to the original version. Discrepancies were discussed until an agreement was reached. This final version was then refined to ensure it is easily understandable.

### Statistical analysis

All data were coded and analyzed using the Statistical Package for Social Sciences (SPSS) version 20 (SPSS Inc., Chicago, IL, USA). All tests were two-tailed. According to the Bayesian interpretations, the chance of replication in future studies is low for *p* values between 0.05 and 0.01, moderate for *p* values between 0.01 and 0.001, and high for *p* < 0.001 [4].

First, descriptive statistics (means, standard deviations, and frequency tables) were calculated for the items and subscales proposed by Costa and McCrae [5]. Second, with the aim of studying the structure of the NEO-PI-3, a confirmatory factorial analysis (CFA), was conducted at the facets level (see below); a targeted rotation of principal components was also evaluated using congruence coefficients with the American normative sample.

Scale reliability was measured by Cronbach’s alpha. For group comparisons, reliability values of 0.7 are considered satisfactory while subscales values approximately 0.6 are considered acceptable [6]. However, it has been argued that internal consistency is less important than retest reliability [7].

The Pearson product–moment correlation method was used to determine the presence or absence of variable correlation. This method was chosen due to its robustness with regards to normality assumptions and for its simple interpretability. For *Pearson*’*s r*, the suggested threshold for effect sizes were *r* = 0.10 = small effect, *r* = 0.24 = medium effect, and *r* = 0.37 = large effect [8].

Sociodemographics groups were compared by ANOVA.

#### Confirmatory factorial analysis

CFA was carried out with the *lavaan* package [9] running in R [10]. The *lavaan* package has been shown to generate the same results as other software packages [11]. Mardia’s kurtosis was used to check for multivariate non-normality: Mardia’s kurtosis = 1,194, *z* = 30.78, *p* < .0001.

Maximum likelihood estimation with robust standard errors and the Satorra–Bentler scaled test statistic were used to test CFA models; this method was chosen because it was unlikely to be affected by deviation from normality in data [12]. Chi square is the traditional fit index used to evaluate an overall model as it assesses the magnitude of discrepancy between the sample and the fitted covariance matrices [13]. However, the use of the chi square test to assess this model fit was found unsatisfactory for a number of reasons [14], including its sensitivity to sample size. The ratio of chi square to the degrees of freedom (*df*) was calculated, with ratios larger than 3 indicating poor fit [15]. Additional parameters for fit estimation were the following: the comparative fit index (CFI), the root mean square error of approximation (RMSEA), and the standardized root mean square residual (SRMR). RMSEA values of 0.08 or lower, SRMR values of 0.09 or lower, and CFI values of 0.90 or higher are considered acceptable [13,16].

Two models were tested, a rather unlikely, unidimensional model, which assumes that all facets load on a single factor, and the *a priori* expected five-factor model, in which all facets were linked to its own latent factor only, the so-called simple structure [17,18]. The more complex models were not tested because they are based on cross-loading (as well as several cross-loadings), which prevents a clear attribution of the predictor to the latent variable it is expected to measure. As a matter of fact, it has been found that increasing the measure’s complexity to comply with the CFA standard led to a reduced convergent and discriminant validity [17].

When CFA failed to reach fit, the orthogonal Procrustes rotation was proposed as a method to test the replicability of the NEO-PI-3 personality factors [18-20]. A dedicated script running in SPSS of the program that performs the orthogonal Procrustes rotation was used to execute the analysis (courtesy of Professor Robert R. McCrae).

According to a shared convention, factor loadings higher than 0.71 (accounting for 50% of variance or more) are considered excellent, 0.63 (40%) very good, 0.55 (30%) good values around 0.45 (20%) fair, and values below 0.32 (10% of variance) poor [21].

Congruence between potentially homologous factors across samples was evaluated using the coefficient of congruence (CC). The CC index ranges from −1.00 (perfect negative similarity) to 1.00 (perfect positive similarity), with zero indicating complete dissimilarity [22]. Reported thresholds for agreements between factors are as follows: very high = 0.90 or above; high = 0.80 to 0.89; and moderate = 0.70 to 0.79 [23].

## Results

The study sample was convenient and somewhat representative of the country’s active population with some overrepresentation of younger ages and clerks (Tables 1 and 2).

**Table 1:** **Composition of the study sample in terms of gender and age in comparison to the general population according to the Greek National Statistics Service for 2009**

**Age group**	**Greek population (approximation for 2009)**	**Study sample**
Total population	11,282,751	734
Males vs. females	48% vs. 52%	40.6% vs. 59.4%
25–29 years old	11.02%	25.81%
29–34 years old	11.31%	12.90%
34–39 years old	10.00%	15.44%
40–44 years old	10.00%	13.13%
44–49 years old	9.21%	10.60%
50–54 years old	8.92%	10.14%
55–59 years old	6.83%	8.29%
60–64 years old	7.09%	3.69%

**Table 2:** **Occupation characteristics of the study sample**

	**Count**	**Percentage**
He/she used to work but is currently unemployed	0	0.00
He/she never worked and neither does now	0	0.00
Clerk (civil or private)	338	63.41
Free professional (tradesman, craftsman)	62	11.63
Doctor, lawyer, engineer, priest, teacher, etc.	75	14.07
Student (college or university)	12	2.25
Blue collar worker (construction worker, farmer)	26	4.88
Housewife	20	3.75
Total	533	100.00

### Internal consistency reliabilities and mean scores for the Greek NEO-PI-3 facets

Mean, standard deviation, skewness, kurtosis, and internal consistency scores (with 95% confidence of interval) for the 30 NEO-PI-3 facets are shown in Table 3.

**Table 3:** **Mean values for the domains and the facets of the Greek NEO-PI-3**

**NEO-PI-3 facet**	**Mean**	**SD**	**Skewness**	**Kurtosis**	**Cronbach’s alpha (95% CI)**	**Cronbach’s alpha (United States)**
Neuroticism	89.06	19.59	0.05	0.19		
N1: Anxiety	17.39	5.05	−0.14	−0.14	0.724 (0.693–0.753)	0.72
N2: Hostility	14.25	4.15	0.24	0.45	0.582 (0.534–0.626)	0.69
N3: Depression	14.07	4.92	0.23	−0.15	0.708 (0.674–0.739)	0.80
N4: Self-consciousness	15.43	3.99	0.21	0.05	0.467 (0.406–0.523)	0.66
N5: Impulsivity	15.48	3.87	0.13	−0.07	0.425 (0.360–0.486)	0.63
N6: Vulnerability	12.44	4.45	0.26	0.27	0.685 (0.649–0.718)	0.70
Extraversion	108.68	15.97	−0.03	−0.15		
E1: Warmth	21.10	4.10	−0.58	1.01	0.648 (0.608–0.685)	0.73
E2: Gregariousness	18.17	4.43	−0.08	−0.13	0.613 (0.569–0.654)	0.77
E3: Assertiveness	15.90	4.14	0.18	0.05	0.589 (0.543–0.633)	0.76
E4: Activity	18.64	3.79	0.10	0.02	0.501 (0.445–0.554)	0.61
E5: Excitement-seeking	15.66	3.96	0.04	0.13	0.445 (0.382–0.503)	0.63
E6: Positive emotions	19.21	4.30	−0.28	0.28	0.636 (0.594–0.674)	0.77
Openness	104.83	16.31	0.27	0.21		
O1: Fantasy	16.07	4.77	0.22	−0.18	0.676 (0.640–0.711)	0.76
O2: Aesthetics	18.02	5.15	−0.07	0.01	0.729 (0.698–0.757)	0.79
O3: Feelings	19.38	3.55	0.06	−0.04	0.437 (0.374–0.497)	0.71
O4: Actions	15.26	3.70	0.12	0.22	0.423 (0.358–0.484)	0.55
O5: Ideas	16.88	4.83	0.07	−0.05	0.694 (0.659–0.726)	0.79
O6: Values	18.82	3.75	0.84	0.17	0.548 (0.497–0.596)	0.69
Agreeableness	116.37	15.82	−0.14	0.11		
A1: Trust	17.23	4.40	−0.31	−0.11	0.651 (0.612–0.688)	0.82
A2: Straightforwardness	20.28	4.31	−0.23	0.05	0.535 (0.483–0.585)	0.67
A3: Altruism	22.39	4.07	−0.59	0.59	0.669 (0.631–0.704)	0.74
A4: Compliance	17.48	4.63	−0.25	−0.13	0.645 (0.605–0.683)	0.68
A5: Modesty	17.82	3.37	−0.08	0.05	0.301 (0.221–375)	0.70
A6: Tender-mindedness	21.18	3.61	−0.57	0.96	0.431 (0.367–0.492)	0.58
Conscientiousness	121.60	19.22	−0.16	−0.09		
C1: Competence	19.86	3.70	−0.14	0.29	0.511 (0.455–0.562)	0.65
C2: Order	19.79	4.94	−0.32	−0.08	0.708 (0.675–0.739)	0.68
C3: Dutifulness	22.79	4.34	−066	0.85	0.655 (0.616–0.692)	0.64
C4: Achievement-striving	20.44	4.16	−0.30	0.05	0.636 (0.595–0.674)	0.73
C5: Self-discipline	20.35	4.32	−0.92	0.01	0.655 (0.616–0.691)	0.81
C6: Deliberation	18.37	4.66	−0.22	−0.03	0.684 (0.648–0.717)	0.70

Most facets exhibited Cronbach’s alpha values above 0.60, the accepted limit of internal consistency reliability for subscales. A few facets exhibited Cronbach’s alpha values lower than 0.50. Overall, the internal consistency reliability measures of the Greek translation were somewhat lower than those observed in the original American sample.

Skewness was always below [3.00] while kurtosis was always below [8.00], indicating that there was no univariate non-normality in the distribution of facet scores.

### Confirmatory factor analysis of the Greek NEO-PI-3

The unidimensional model was rejected on the basis of the fit indexes: chi square = 4,975.31, *df* = 405, *p* < 0.0001; CFI = 0.387; RMSEA = 0.124 (95%CI: 0.121–0.127); SRMR = 0.137.

The *a priori* expected five-factor model had a better fit for all indexes (Table 4).

**Table 4:** **Confirmatory factor analysis of the facets of the Greek NEO-PI-3**

	**Neuroticism**	**Extraversion**	**Openness to experience**	**Agreeableness**	**Conscientiousness**
N1: Anxiety	0.724				
N2: Hostility	0.638				
N3: Depression	0.823				
N4: Self-consciousness	0.606				
N5: Impulsivity	0.466				
N6: Vulnerability	0.738				
E1: Warmth		0.723			
E2: Gregariousness		0.571			
E3: Assertiveness		0.332			
E4: Activity		0.457			
E5: Excitement-seeking		0.415			
E6: Positive emotions		0.691			
O1: Fantasy			0.480		
O2: Aesthetics			0.714		
O3: Feelings			0.567		
O4: Actions			0.422		
O5: Ideas			0.681		
O6: Values			0.435		
A1: Trust				0.359	
A2: Straightforwardness				0.496	
A3: Altruism				0.874	
A4: Compliance				0.408	
A5: Modesty				0.282	
A6: Tender-mindedness				0.675	
C1: Competence					0.665
C2: Order					0.536
C3: Dutifulness					0.713
C4: Achievement-striving					0.706
C5: Self-discipline					0.799
C6: Deliberation					0.603
Estimated Cronbach’s alpha	.830	.717	.723	.719	.826
	Robust chi square	Chi square/*df*	CFI	RMSEA (90%CI)	SRMR
Expected	p > .05	<3	> .900	<.08 (<.08)	<.09
Observed	3,241.44, *df* = 395, *p* < .0001	8	.618	.099 (.096–.102)	.119

Overall, the fit was still poor. However, loading of the facets on their own hypothesized factors was acceptable, and the estimated Cronbach’s alphas for the hypothesized factors were very good.

### Procrustes rotation analysis of the Greek NEO-PI-3

The Procrustes rotation analysis revealed a good replication of the expected five-factor structure of the NEO-PI-3.

The loading of the facets on their own factors was good to excellent with few exceptions (Table 5).

**Table 5:** **Factor loadings for Greek NEO-PI-3 facet scales after Procrustes rotation**

	**Factor**					**VCC**
**NEO-PI-3 facet**	**N**	**E**	**O**	**A**	**C**	
N1: Anxiety	*.80*	–.08	–.05	.00	–.02	.99^a^
N2: Angry hostility	*.68*	–.04	–.13	*–.41*	–.06	.98^a^
N3: Depression	*.80*	–.24	–.03	.05	–.15	.97^a^
N4: Self-consciousness	*.67*	–.16	–.16	.18	–.06	.97^a^
N5: Impulsiveness	*.56*	.34	.06	–.35	–.26	.98^a^
N6: Vulnerability	*.59*	–.24	–.09	–.08	*–.49*	.96^a^
E1: Warmth	–.16	*.61*	.11	*.47*	.21	.98^a^
E2: Gregariousness	–.32	*.54*	.07	.14	.00	.96^a^
E3: Assertiveness	–.34	.38	.07	*–.51*	.27	.94^a^
E4: Activity	–.06	*.53*	–.03	–.15	*.47*	.94^a^
E5: Excitement-seeking	.02	*.52*	*.43*	–.23	–.06	.87^b^
E6: Positive emotions	–.25	*.71*	.22	.12	–.05	.95^a^
O1: Fantasy	.12	.18	*.58*	–.14	–.34	.99^a^
O2: Aesthetics	.14	.08	*.76*	.12	.09	.99^a^
O3: Feelings	.28	*.44*	*.51*	.04	.22	.98^a^
O4: Actions	–.23	.08	*.53*	–.20	–.07	.90^b^
O5: Ideas	–.14	.00	*.78*	–.01	.15	.99^a^
O6: Values	–.06	.09	*.55*	–.02	–.03	.96^a^
A1: Trust	–.27	.33	–.05	*.51*	–.11	.92^b^
A2: Straightforwardness	–.06	–.03	–.04	*.64*	.19	.98^a^
A3: Altruism	.02	.34	.06	*.59*	*.46*	.93^b^
A4: Compliance	–.23	–.14	–.07	*.73*	–.02	.99^a^
A5: Modesty	.23	–.09	–.17	*.48*	.10	.95^a^
A6: Tender-mindedness	.13	.32	.05	*.57*	.32	.88^b^
C1: Competence	–.29	.25	.07	–.03	*.65*	.98^a^
C2: Order	.02	–.08	–.04	.07	*.65*	.95^a^
C3: Dutifulness	.03	.21	.01	.39	*.68*	.90^b^
C4: Achievement-striving	–.08	.29	.06	–.05	*.75*	.99^a^
C5: Self-discipline	–.29	.07	.00	.15	*.74*	.98^a^
C6: Deliberation	–.24	–.30	.00	.25	*.66*	.99^a^
Congruence^c^	.97^a^	.96^a^	.94^a^	.96^a^	.96^a^	.96^a^

Note. *N* = 734. These are principal components rotated to the American normative target (Costa and McCrae, [5]). Loadings greater than .40 in absolute magnitude are given in boldface type. Highest loadings are marked in italics.

*Abbreviations: NEO-PI-3* NEO Personality Inventory-3, *N* Neuroticism, *E* Extraversion, *O* Openness, *A* Agreeableness, *C* Conscientiousness, *VCC* variable congruence coefficient.

^a^Congruence higher than that of 99% of rotations from random data; ^b^Congruence higher than that of 95% of rotations from random data; ^c^Factor/total congruence coefficient with target matrix.

Only a minority of facets also loaded on a different factor than their own with an absolute factor loading higher than 0.40.

CC values for potentially homologous factors across samples were within high to very high interval. The extracted factors in the Greek sample can be considered reasonably homologous to their counterparts in the American normative sample.

### Scores on the five dimensions of the Greek NEO-PI-3

The pattern of raw mean scores is similar to that seen in the US and elsewhere (Table 6).

**Table 6:** **Mean values and correlations for the big five factors of the Greek NEO-PI-3**

	**Mean (95% CI)**	**Neuroticism**	**Extraversion**	**Openness to experience**	**Agreeableness**
Neuroticism	89.06 (87.64–90.48)				
Extraversion	108.68 (107.52–109.84)	−0.546*			
Openness	104.43 (103.21–105.66)	−0.108**	0.466*		
Agreeableness	116.37 (115.23–117.52)	−0.255*	0.545*	0.130**	
Conscientiousness	121.60 (120.20–122.99)	−0.506*	0.457*	0.113**	0.635*

*p < .0001; **p < .05.

As expected, Neuroticism was negatively related to the other factors, Extraversion was positively related to Openness, Agreeableness, and Conscientiousness, and Agreeableness was positively related to Conscientiousness. The links between Openness and Agreeableness or Conscientiousness were less evident but in the expected direction. Correlation between factors was never so high as to prevent discriminant validity.

### Differences by gender, age, and education on the Greek NEO-PI-3

Females scored higher than males on the Neuroticism and the Openness factors. Males scored marginally higher than females on the Conscientiousness factor (Table 7).

**Table 7:** **Differences by gender (females–males, after standardizing the scores as** ***z*** **-scores using the total samples M and SD) and correlation with age and education on the Greek NEO-PI-3**

	***d*** **sex**	***r***	
		**Age**	**Education**
N1: Anxiety	.57	–.05	–.01
N2: Angry hostility	.25	.01	–.01
N3: Depression	.39	.02	–.04
N4: Self-consciousness	.30	.05	–.12
N5: Impulsiveness	.30	–.13	.02
N6: Vulnerability	.52	–.03	–.03
E1: Warmth	–.06	.05	–.05
E2: Gregariousness	.13	–.10	–.01
E3: Assertiveness	–.29	–.05	.09
E4: Activity	–.01	.04	–.04
E5: Excitement-seeking	–.18	–.30	.09
E6: Positive emotions	.00	–.17	.03
O1: Fantasy	.14	–.29	.14
O2: Aesthetics	.20	–.07	.21
O3: Feelings	.26	–.16	.18
O4: Actions	.14	–.23	.16
O5: Ideas	.00	–.17	.23
O6: Values	.08	–.24	.26
A1: Trust	–.09	.16	–.02
A2: Straightforwardness	.14	.08	.01
A3: Altruism	–.01	.11	–.08
A4: Compliance	.06	.20	–.09
A5: Modesty	.03	.15	–.06
A6: Tender-mindedness	.03	.09	–.01
C1: Competence	–.31	.07	.05
C2: Order	.06	.07	–.02
C3: Dutifulness	.00	.09	.00
C4: Achievement-striving	–.21	–.02	.05
C5: Self-discipline	–.16	.09	–.01
C6: Deliberation	–.15	.13	–.01

Overall, the pattern of gender differences is similar to what one sees around the world, except that Greek females did not score higher than males on Agreeableness.

Age and education were modestly related to Greek NEO-PI-3 facets.

## Discussion

The current paper reports on the results of the Greek translation of the NEO-PI-3. Most facets exhibited Cronbach’s alpha values above 0.60, though overall, the internal consistency reliability measures of the Greek translation were lower than those observed in the original American sample. Confirmatory factor analysis failed to reach the predefined fit. However, it showed acceptable loading of the facets on their own hypothesized factors and very good estimations of Cronbach’s alphas for these factors; therefore, it partly supports the five-factor structure of the NEO-PI-3. Principle components after Procrustes rotation closely resembled the factors of the American normative sample. Correlations between dimensions were as expected and similar to those reported in the literature.

The literature suggests that, overall, the psychometric properties of NEO-PI-R scales have been found to generalize across ages, cultures, and methods of measurement [7].

The internal consistency originally reported for both NEO-PI-R domains (*N* = 0.92, *E* = 0.89, *O* = 0.87, *A* = 0.86, *C* = 0.90) as well as facets (0.56–0.81) was high. The internal consistency of the NEO-PI-3 was similar to that of the NEO-PI-R, with alphas ranging from 0.89–0.93 for domains and 0.54–0.83 for facets [24,25]. The literature appears to support the internal consistencies listed in the manual. The Filipino translation of the NEO-PI-R has internal consistency of domain scores ranging from 0.78–0.90 [26], with facet alphas having a median of 0.61 [27].

Test-retest reliability (administered 3 months later) of an early version of the NEO-PI domains was *N* = 0.87, *E* = 0.91, and *O* = 0.86 [28]. The test-retest reliability reported in the manual of the NEO-PI-R over 6 years was *N* = 0.83, *E* = 0.82, *O* = 0.83, *A* = 0.63, and *C* = 0.79. Costa and McCrae pointed out that this not only shows good reliability of the domains but also that they are stable over long periods of time (past the age of 30), as the scores more than 6 years apart were only marginally different from the scores measured a few months apart [5]. Other research has also shown acceptable test-retest reliability. A 2001 study by Kurtz and Parrish on the short-term test-retest reliability yielded alpha coefficients 0.9–0.93 for domains and 0.70–0.91 for facets after a 1-week interval [29]. A 2006 study by Terracciano et al. [30] on long-term test-retest reliability yielded alpha coefficients 0.78–0.85 for domains and 0.57–0.82 for facets after a 10-year interval.

In terms of criterion validity, Conard (2006) found that Conscientiousness significantly predicted the GPA (grade point average) of college students, more so than by using Scholastic Assessment Test (SAT) scores alone [31]. Garcia et al. correlated a Spanish version of the NEO to predictors of teacher burnout in Sevilla, Spain. Neuroticism was related to the ‘emotional exhaustion’ factor of burnout with a correlation coefficient equal to 0.44. Agreeableness related to the ‘personal accomplishment’ factor of burnout (which is negatively scored when predicting burnout) exhibited a score of *r* = 0.36 [32]. A group of authors in 2006 found that in a minority students population, the Extraversion trait was correlated to Career Decision Making Self-Efficacy (CDMSE) with *r* = 0.30, while Neuroticism was strongly related to Career Commitment after controlling for CDMSE (*r* = 0.42) [33]. Finally, in 2007, Korukonda reported that Neuroticism was positively related to computer anxiety, while Openness and Agreeableness were negatively related to each other [34].

Cross-cultural stability of an instrument can be considered evidence of its validity. A huge amount of cross-cultural research has been carried out on the Five-Factor model of personality by utilizing the NEO-PI-R and its shorter version, the NEO-FFI. A collection of selected papers from various researchers across the globe have been presented covering various issues in cross-cultural research on the FFM [35]. This monograph has also presented data for the FFM from several cultures. The robustness of the FFM has been proven across different cultures; these include but are not limited to the following: Chinese [36,37], Estonian and Finnish [38], Filipino and French [39], Indian [40], Portuguese [41], Russian [42], South Korean [43], Turkish [44], Vietnamese [45], sub-Saharan cultures like Zimbabwean [46], Austrian, former East and West German, and Switzerland’s culture [47]. On the basis of the data from 16 cultures, it has been suggested that the concepts of Neuroticism, Openness, and Conscientiousness are cross-culturally valid, while Extraversion and Agreeableness are components of interpersonal circumflex and are more sensitive to cultural context [48]. It is interesting to note that in the Zuckerman five-factor model ‘Openness to experience’ is deliberately excluded because Zuckerman suggested that it does not meet the criteria for a truly ‘basic’ factor of personality [49]. Furthermore, it seems that the age differences in the five factors of personality across the adult life span are paralleled in samples from Germany, Italy, Portugal, Croatia, and South Korea [50]. The age and gender differences and fluctuations found in the original American sample [3] were generally confirmed in an analysis of the data from 51 cultures [51-53]. These findings are paralleled by the results of the current Greek validation study.

In conclusion, we submit that the results of the current study confirm the reliability of the Greek translation and adaptation of the NEO-PI-3. The inventory has comparable psychometric properties in its Greek version as in the original and other national versions, although with somewhat lower values, and it is suitable for clinical as well as research use in Greek speaking populations.
